# Projecting Labour Market Imbalances and Skill Mismatch Under Demographic Change in the EU

**DOI:** 10.1007/s10680-025-09758-2

**Published:** 2025-12-04

**Authors:** Guillaume Marois, Michaela Potančoková, Agnieszka Bezat, Jesús Crespo Cuaresma

**Affiliations:** 1 International Institute for Applied Systems Analysis, Wittgenstein Centre for Demography and Global Human Capital (IIASA, VID/OeAW, University of Vienna), Laxenburg, Austria; 2 Asian Demographic Research Institute, School of Sociology and Political Sciences, Shanghai University, Shanghai, China; 3 Department of Quantitative Methods & Information Technology, Kozminski University, Warsaw, Poland; 4 Department of Economics, Vienna University of Economics and Business (WU), Vienna, Austria

**Keywords:** Labour mismatches, Occupation, Projection, Europe, Skills, Shortage

## Abstract

We assess long-term labour mismatches in the European Union (EU27) by projecting the occupational distribution of workers and skill-specific labour demand up to 2060. Using a dynamic microsimulation approach (*Link4Skills-Mic*), we jointly model demographic, educational, and labour force dynamics at the individual level and combine country-specific projections of labour supply with projections of occupational demand. The analysis highlights growing imbalances: although the supply of highly educated workers continues to rise, shifts in demand are not evenly distributed across skill levels. Consequently, underutilization of high-skilled workers is projected to coexist with persistent vacancies in medium- and low-skilled occupations. Rather than indicating widespread labour shortages, these trends point to structural mismatches driven by the misalignment of worker qualifications, job characteristics, and hiring practices. To explore potential responses, we examine a series of policy scenarios such as expanded immigration, education reform, mid-career retraining, delayed retirement, and employer-led automation and upskilling. The findings show that, while certain policies can reduce specific mismatches, no single intervention closes all the gaps that emerge. Notably, automation reduces vacancies but increases underutilization, whereas human capital strategies shift mismatches across skill levels. These results suggest that addressing future labour mismatches will require coordinated, comprehensive and varied strategies that integrate demographic realities with evolving job demands in Europe’s ageing and increasingly digitalized and knowledge-based economies.

**Supplementary Information:**

The online version contains supplementary material available at 10.1007/s10680-025-09758-2.

## Introduction

Europe is undergoing significant demographic changes that are expected to reshape its socio-economic landscape in the coming decades. The population is aging rapidly, leading to an increase in the proportion of older adults and a shrinking working age population. As the working age population declines, policymakers and politicians are worried about potential labour shortage (Pouliakas et al., 2024). The strain on social support systems intensifies, while the need for a dynamic and adaptable workforce grows.

Simultaneously, structural shifts in the economy driven by technological advancements are increasing the demand for highly skilled workers (CEDEFOP, 2016; Goos et al., 2019). In most European countries, younger generations who enter the workforce have higher educational attainment than those who retire (Lutz et al., 2018). However, this growing supply of better educated and presumably also better qualifiedworkers does not always align with the demand for specific skills in the labour market, leading to potential mismatches (Pouliakas et al., 2024).

In public discourse, the claims of future labour and skills shortages tend to be grounded in projected working age population declines which are expected to exacerbate labour shortages. Such claims are often based on population projection models that do not explicitly take into account labour force dynamics by educational level and other important workforce characteristics. To date, official population projections of the European Union have focused on the age and sex distribution of the population, providing insights into the broad demographic trends in the future (Eurostat, 2023). A limited number of population projections producers extend the standard age and sex dimensions by educational composition and/or by labour force participation (Fuchs et al., 2018; Loichinger, 2015; Marois et al., 2020). Such multidimensional projections are necessary for the assessments of future labour supply because labour force activity is closely correlated with educational attainment, age and sex. However, these labour supply models do not extend the modelling of the occupation of workers as a core dimension of the analysis. Including occupation into population projections allows us to gain valuable quantitative insights of future labour mismatches.

To anticipate the extent of the potential future mismatches in the labour markets of EU countries, it is necessary to employ projection exercises that model labour supply and labour demand simultaneously. Most existing occupational forecasts integrate econometric labour supply and demand modules within general equilibrium (GE) frameworks, treating demographic dynamics as exogenous (OECD, 2019). In turn, input–output models infer labour demand from sectoral output while treating labour supply as exogenous and assuming largely static relationships (Miao et al., 2024). CEDEFOP’s skills forecast (CEDEFOP, 2023) is based on a modular modelling framework which integrates a macroeconomic labour demand projection model with a labour supply model. This specification extends the cohort-component population projection by age and sex (EUROPOP by EUROSTAT) by including education, labour force participation and employment rates and uses a time series model based on Labour Force Survey (LFS) data. In the context of this methodological approach, educational trends do not interact with demographic trends and the labour supply and demand models must be manually aligned.

To overcome the limitations of separating demographic, educational and labour market processes, we adopt a multidimensional demographic modelling approach to joint modelling of labour supply and demand. We employ a dynamic microsimulation model, *Link4Skills-Mic,* that simultaneously captures demographic, educational, and labour force dynamics. Multidimensional microsimulation models are well suited to incorporate several dimensions of heterogeneity that influence labour force outcomes and to interlink labour supply and labour demand in a single framework. They also provide an alternative and complementary view to econometric approaches, highlighting particular causal mechanisms that may be of importance to design policy.

We project the distribution of the occupation of workers in the European Union (EU27) for the period 2020–2060 and explore how the interplay between future demographic change and continuing educational expansion will affect the occupational distribution of the EU workforce over the coming decades. The model incorporates the relationship between demographic dynamics, educational expansion and the demand for high-skilled, medium-skilled, and low-skilled jobs.

We analyse how the long-term trends in the changing composition and characteristics of workers interact with projected future skill demand. Labour shortages and mismatches indicate inefficiencies in the labour markets, revealing imbalances between the labour supply and demand for specific skills and qualifications. The gap between the total labour demand (defined as all jobs in the economy) and occupied jobs by skill levels projected in this study points to potential labour and skill shortages, although the underlying causes should be interpreted with caution. Alignment or misalignment of workers skills can result in skill mismatch or underutilisation of workers. In this analysis, we define underutilized workers as those who remain unemployed or who occupy lower-skilled jobs than those corresponding to their educational qualifications.

Long-term planning of labour market and education policies in increasingly knowledge-based economies requires projections intersecting demographic, educational and labour demand trends. This study provides the first long-term projection of labour force by occupational skill levels with multiple scenarios that outline several policy options for adapting to future demographic challenges. While most existing econometric projections are limited to the medium term, long-term analysis is essential to capture the effects of population momentum and workforce ageing. For instance, CEDEFOP’s skill forecast (CEDEFOP, 2023) presents a single scenario whose projection horizon ends in 2035. Our approach addresses this gap by leveraging the flexibility of microsimulation to model "what-if" scenarios over a longer horizon, ranging up to 2060. We consider how an ageing but increasingly educated labour supply interacts with macro-level changes such as deindustrialization, automation, digitalisation, and sectoral shifts. The scenarios assess the impact of various policy responses (including migration policy, education reform, and employer-driven automation) on future job vacancies and labour market mismatches. In doing so, our study provides empirical evidence and insights on how to respond to the concern expressed by the European Commission and OECD about a widening skills gap, driven by slow adaptation in education systems and underinvestment in lifelong learning (European Commission, 2020; OECD, 2021).

To understand the forces driving current and future skill mismatches, we first examine the structural transformations reshaping labour markets, namely: technological advancement, demographic trends, and sectoral shifts. These forces create the context within which our microsimulation model operates. The structure of Link4Skills-mic, the underlying assumptions and the modelling of changes in occupational skill levels and skill-specific labour demand are described in Sect. 3 and further detailed in the Supplementary Material. Section 4.1 summarises results of the reference scenario for aggregate EU27 and Sect. 4.2 highlights how alternative scenarios of migration, human capital development and employer responses impact projected skill composition and worker underutilisation. In Sect. 5, we conclude with a discussion of these finding and present the limitations of our modelling framework.

## Structural Economic Transformations and Labour Market Mismatches

Examining economic structural change is a fundamental requirement for understanding the dynamic nature of modern economies (Acemoglu & Robinson, 2013; Kuznets, 1973). The transformation of economic structures, whether through shift in sectoral composition (Baumol, 1967; Hartwig & Krämer, 2023; McMillan & Rodrik, 2011), technological advancements (Acemoglu & Restrepo, 2019; Brynjolfsson & McAfee, 2014) or demographic changes (Bloom & Canning, 2008; Filmer & Fox, 2014; Lee & Mason, 2010), influence labour market dynamics, not only creating new employment opportunities but also exacerbating labour mismatches, underutilization, and shortages when workers’ existing skills fail to align with shifting sectoral demand (Goldin & Katz, 2008). Understanding the interplay between these forces is crucial for designing policy interventions that mitigate structural inefficiencies and ensure labour market adaptability.

Technological change not only increases productivity in certain industries but also reshapes sectoral employment demand by displacing routine jobs and creating new skill-intensive occupations (Acemoglu & Restrepo, 2019). As economies develop, resources often shift towards sectors with lower productivity growth, leading to slower overall economic growth (Baumol, 1967; Hartwig & Krämer, 2023). As a consequence, resource shifts within and across sectors are required to achieve productivity gains (McMillan & Rodrik, 2011). The implications of these trends are already visible in high-income economies, where deindustrialization and automation have led to declining demand for middle-skilled workers, leading to polarization in the labour market (Autor et al., 2013).

Endogenous growth models highlight that the shift toward knowledge-based economies necessitates an analytical framework that integrates technological innovation with macroeconomic labour trends (Romer, 1990). The work by Acemoglu and Restrepo (2019) examines how automation and artificial intelligence reshape labour markets, highlighting the need for policy interventions to address potential inequalities. Brynjolfsson and McAfee (2014) underscore how digitalization and automation are reshaping economic structures by altering the demand for labour, capital, and skill sets. The authors argue that automation, by displacing routine jobs and rendering many traditional skills obsolete, increases structural unemployment—particularly among medium-skilled workers—while simultaneously creating high-skill opportunities in digital and knowledge-based industries. This transformation contributes to job vacancies, potential skill mismatches, and wage suppression in certain sectors (Acemoglu & Restrepo, 2019; Autor et al., 2013; Brynjolfsson & McAfee, 2014).

Demographic changes further compound labour market mismatches by influencing both the supply and demand for labour. In this context, researchers analyse the economic implications of aging populations on labour markets and growth, underscoring the need for adaptive policies to mitigate potential negative effects (Bloom & Canning, 2008) and emphasizing the increased dependency ratios and potential strain on public resources (Lee & Mason, 2010; Marois et al., 2020). Aging societies, particularly in Europe and East Asia, are at risk of increasing labour shortages, prompting policy discussions on automation, immigration, and pension reform (Bloom et al., 2010). Ageing populations in many advanced economies (e.g., Japan, Germany) have led to shrinking workforces and unfilled vacancies in key industries (Bloom et al., 2010), as the supply of younger workers remains insufficient to replace retirees (Bloom & Canning, 2008).

At the same time, evolving work preferences—exemplified by the rise of the gig economy—have altered traditional employment patterns, leading to an increase in non-standard work arrangements (Wood et al., 2019). Demographic trends, in conjunction with sectoral and technological shifts, demonstrate that labour mismatches are not driven by a single factor but by the interaction of multiple structural forces shaping the workforce.

Demographic changes further complicate labour market dynamics, particularly in advanced economies facing an aging workforce. The projections in CEDEFOP (2023) indicate that many EU member states, particularly Germany, Italy, and Eastern European countries, will experience significant labour force declines over the next decade due to low birth rates and increasing retirement rates. Similarly, the Bureau of Labor Statistics projects that the share of older workers will continue to rise in the U.S., putting pressure on sectors such as healthcare, social services, and education (Bureau of Labor Statistics, 2024).

While high-skilled professions are expanding, medium-skilled occupations—traditionally in manufacturing, clerical work, and routine administrative tasks—are increasingly at risk. Studies suggest that routine-intensive sectors such as transportation, customer service, and financial administration are experiencing rapid employment declines due to automation (Bureau of Labor Statistics, 2024; CEDEFOP, 2023). Routine-based employment is declining in both the U.S. and Europe, as companies invest in labour-saving technologies to increase efficiency and reduce costs (Autor et al., 2013; CEDEFOP, 2023). Conversely, technological developments are expected to generate innovations that augment human labour in numerous fields, thereby creating new jobs that require advanced cognitive skills. The consequences of this polarization extend beyond job losses. The World Economic Forum’s Future of Jobs Report (WEF, 2025) highlights that while technology is creating new employment opportunities, it is also deepening wage disparities between high-skilled professionals and those engaged in low-wage service work, such as retail, food service, and personal care. Investment in vocational training and digital education will be critical in ensuring that technological progress does not lead to widespread unemployment and social disruption (WEF, 2025). The future of work is thus expected to depend on the ability of governments to implement proactive reskilling initiatives, education reform, and better alignment between labour supply and industry needs. Workers in at-risk occupations must transition into emerging fields, such as green energy, AI development, and healthcare, where demand is expected to grow (WEF, 2025). Without such interventions, structural imbalances in the labour market may persist, leading to prolonged economic inefficiencies and constraints on long-term growth.

The existing literature on labour market transformations offers valuable insights into the nature of structural changes driven by technological advancements, demographic shifts, and sectoral reallocation. However, much of the research remains fragmented, often analysing individual aspects in isolation without systematically linking demographic trends to occupational structures and skill mismatches (Acemoglu & Restrepo, 2019; Bloom & Canning, 2008). While studies highlight skill shortages and underutilization (Autor et al., 2013; Goldin & Katz, 2008), they provide limited empirical evidence on the effectiveness of migration policies, or employer-driven training (Rodrik, 2016).

We address these shortcomings by developing a microsimulation-based forecasting approach that integrates demographic variables with occupational demand, allowing for a dynamic assessment of skill mismatches and labour shortages. By simulating the effects of policy interventions, such as migration strategies and upskilling programs, this piece of research bridges the gap between labour market projections and evidence-based policy solutions.

## Methodology: A Microsimulation Model for Occupation Projections

### The Basics of the Microsimulation Model: Demography, Education and Labour Force Participation

The novel methodological contribution of our modelling exercise lies in the extension of existing microsimulation frameworks by introducing an occupation module that is used to project the distribution of workers across occupational skill groups. Given the large number of states required to model demographics, education, labour force participation, migration, and occupational choice jointly, a multistate approach would be impractical, making microsimulation a more feasible and flexible solution. We develop this modelling framework under the name *Link4Skills-Mic*.

The underlying modelling structure and its demographic, labour force, and education modules, together with their assumptions, are described exhaustively in Supplementary Information S1. These modules follow the microsimulation framework developed in the QuantMig-Mic model^1^ (Marois et al., 2023), which allows for the analysis of heterogeneous populations in terms of demographic and socio-economic characteristics (age, sex, education and labour force participation), as well as immigration-related variables (region of birth, duration of residence, and age at immigration). Demographic events are simulated at the individual level using a Monte Carlo process.

The main quantitative assumptions of the demographic, education, and labour force modules are shown in Table 1. The column “Dimensions of heterogeneity” specifies the set of individual characteristics used as predictors for each one of the events modeled, providing a concise overview of how demographic, educational, and labour force components are interlinked in the microsimulation. The modules are interconnected through sequential causal relationships. Demographic processes (fertility, mortality, and migration) determine the population structure by age, sex, and origin, which in turn shapes the pool of individuals eligible for labour market participation. Educational attainment influences demographic behaviour, labour force participation probabilities, and occupational outcomes. The occupation module described in Sect. 3.2 is applied to the active population, allocating individuals across unemployment and three broad skill groups. In this way, demographic dynamics affect labour supply, while labour demand, which is linked to population size and economic structure, constrains occupational outcomes. The interaction of these modules allows the model to capture how demographic and educational changes jointly drive the composition of the labour market over time.

**Table 1:** Main projection assumptions

Events	Summary of the assumptions	Dimensions of heterogeneity	Source
Fertility	Total fertility rate (unweighted country average): 2020–2024: 1.51 2035–2039: 1.58 2055–2059: 1.64	Country of residence, age, education	SSP2 scenario, Wittgenstein Centre Human Capital Data Explorer (KC et al., 2024)
Mortality	Life expectancy at birth (unweighted country average) (female/male) 2020–2024: 83.5/77.6 2035–2039: 87.0/81.4 2055–2059: 91.2/85.6	Country of residence, age, sex, education	SSP2 scenario, Wittgenstein Centre Human Capital Data Explorer (KC et al., 2024)
International migration Non EU/EFTA	Immigrants (5-year): 2020–2024: 15,272,419 2035–2039: 12,726,958 2055–2059: 12,395,784 Emigrants (5-year): 2020–2024: 7,279,140 2035–2039: 6,838,362 2055–2059: 6,910,837	Immigrants: Country of destination, region of origin, age, sex, education Emigrants: Country of residence, age, place of birth (native, other EU country, non-EU)	Marois et al. (2023), based on Aristotelous et al. (2022)
Intra-EU migration	Average rates of 2011–2019 (OD matrix) by place of birth (native, other EU country, non-EU) For the outmigration rate of native and other EU born, convergence by 2050 to the average rate of countries with positive net intra-European migration in 2011–2019	Country of residence, country of destination, age, place of birth (native, other EU country, non-EU)	Marois et al. (2023), based on Aristotelous et al. (2022)
Education	Educational attainment at age 30–34 (Low/Medium/High) 2020: 12.96%/47.88%/39.15% 2040: 9.39%/41.71%/48.91% 2060: 7.74%/39.75%/52.50%	Cohort, age, sex, education of the mother, country of birth	Marois et al. (2023), based on Marois et al. (2019a)
Labour force participation	Labour force participation rates (age 15–24/25–54/55–74) 2020: 38.6%/85.3%/36.3% 2040: 38.9%/87.2%/39.9% 2060: 36.2%/85.5%/42.5%	Cohort, age, sex, education, country of residence, region of birth, duration of stay, age at immigration	Marois et al. (2023), based on Marois et al. (2019b)

The assumptions for fertility and mortality are based on the reference scenario (SSP2) of the Wittgenstein Center’s human capital population projections (KC et al., 2024). The Shared Socio-economic Pathways (SSP) scenario narratives describe how future societies and economies could evolve in response to different levels climate change adaptation and mitigation during the twenty-first century. SSP2 is the “middle-of-the-road” scenario assuming continuation of past fertility, mortality and migration trends under moderate levels of human development, and it integrates recent short-term disruptions linked to the COVID-19 pandemic including the continuing fertility declined across European societies. The assumption setting is informed by international expert polls which provide consistent assumptions on fertility and mortality across countries. Migration rates between EU countries are derived from the bilateral migration flows by Aristotelous et al. (2022) for the period 2011–2019, and projected assuming long-term convergence in emigration rates towards the average of major destination countries in western and northern Europe. The overall immigration flow from the rest of the world regions towards whole EU27 is derived by applying average emigration rate for the period 2011–2019 from Aristotelous et al. (2022) to the projected population sizes in the source regions of the world from the Wittgenstein Center world projection (KC et al., 2024), resulting in an increase in migration flows from demographically young regions such as Sub-Saharan Africa and West Asia. Immigration flow for the period 2020–2024 is adjusted to account for the inflow of Ukrainians at the onset of the war in 2022. These lump sum inflows for the whole EU are further distributed towards the respective destination countries using historical patterns. We also assume that the educational attainment of future immigrants follows the cohort trend of recent immigrants from the same world region. Taken together, these demographic assumptions yield outcomes that are comparable to the EUROPOP 2023 (Eurostat, 2023). The deviations mainly reflect discrepancies in migration assumptions.

Using parameters obtained from the estimation of an ordered logit model, the education module assigns the highest level of education attained over the life course based on historical country and sex-specific secular trends, and considering the intergenerational transmission of education. The education variable is classified into three broad categories based on the International Standard Classification of Education (ISCED, low education: none or ISCED 1–2, i.e. lower secondary or below; medium education: ISCED 3, i.e. completed upper secondary, and high education: ISCED 4 or higher, i.e. post-secondary non-university or tertiary–university–education). The projection assumptions result in a continuation of past trends of increasing educational attainment for the EU as a whole in the future, with around 53% of the cohort born in 2030–2034 attaining post-secondary education, compared to 39% for the cohort born in 1990–1994.

The labour force participation module determines whether individuals are in or out of the labour force based on their socio-demographic characteristics. The participation status is superimposed on the population estimates, with future labour force participation rates by age, sex, country and education estimated using the cohort-development approach, after controlling for the impact of immigration. By including variables related to the migration status of individuals in the logistic regression specification used for the projection exercise, we take into account that labour force integration processes vary by place of birth (see Table S1 in the Supplementary Information, which provides the corresponding parameter values). Other things being equal, immigrants from certain regions, such as North Africa and the Middle East, have lower labour force participation rates, especially among women and recent arrivals (persons who migrated within the last five years). This can be explained by a variety of linguistic and cultural barriers, the lack of recognition of diplomas, smaller professional networks, and degrees that provide less applicable skills in the European labour market (Bevelander, 2005; Büchel & Frick, 2005; Kahn, 2004; Model & Lin, 2002; OECD, 2010).

### Projecting the Occupational Distribution of Workers

Link4Skills-mic incorporates a module to project the occupational skill levels of workers, conditional on active labour force participation. In addition to the unemployed category, the occupation variable is categorized into three broad occupational groups based on the International Standard Classification of Occupations (ISCO): high-skill job (ISCO 1—Managers; 2—Professionals; 3—Technicians and Associate Professionals); medium-skill job (ISCO 4—Clerical Support Workers; 5—Service and Sales Workers; 6—Skilled Agricultural Forestry and Fishery Workers; 7—Craft and Related Trades Workers; 8—Plant and Machine Operators, and Assemblers). and low-skill job (ISCO 9—Elementary Occupations). These groups roughly correspond to the skills associated with the three levels of education included in the model.

The occupation module is overlaid to the labour force module and its output is assumed not to influence demographic dynamics through any feedback. While the total number of active workers is determined by demographic, educational, and participation processes, the occupation module allocates these workers across occupations based on their socioeconomic characteristics and the skill-specific labour demand, with population size driving aggregate demand.

The modelling framework assumes that the probability that individual *i* works in occupation *k* (of *K* possible ones) can be expressed through a multinomial logit model, so that,

1 $$\begin{aligned} ln\frac{\text{Pr}\left({Y}_{i} = k\right)}{\text{Pr}\left({Y}_{i}=K\right)}&={\beta }_{0k}+{\beta }_{1k}{SEX }_{i}+{\beta }_{2k}{EDU}_{i} \\ & \quad +{\beta }_{3k}{SEX}_{i}{EDU}_{i}+{\beta }_{4k}{AGEGR}_{i}+{\beta }_{5k}{{AGEGR}^{2}}_{i} \\ & \quad +{\beta }_{6k}{IMMVAR}_{i}+{\beta }_{7k}{IMMVAR}_{i}{EDU}_{i}\\ & \quad+{\beta }_{8k}{IMMVAR}_{i}{SEX}_{i}+{\lambda }_{1k}\left(\frac{{DemH}_{c,t}}{{LF}_{c,t}}\right)\\ & \quad+{\lambda }_{2k}\left(\frac{{DemM}_{c,t}+{DemL}_{c,t}}{{LF}_{c,t}}\right)+ {\varepsilon }_{ik}\end{aligned}$$

The parameters of the model in Eq. (1) are estimated by maximum likelihood using the Newton–Raphson iterative algorithm with a generalized logit link function. The estimation uses the data from the EU-Labour Force Survey (Eurostat, 2024) spanning the period 2014–2022, combined with the country-specific time series of labour demand estimates by occupation provided by CEDEFOP (2023).^2^ The years 2020 and 2021 are excluded from the sample, to remove the temporal distortion of the COVID pandemic on labour force participation and employment. We include in the modelling only respondents who were in the labour force (including unemployed individuals) and had no missing values in any of the predictors. The particular specification used was selected after a model selection step based on the estimation of models employing different sets of variable interactions and different modulations of the supply of workers and labour demand variables. All model parameter estimates are statistically significant at any reasonable significance level. The model fit statistics can be found in Table S2.

**Fig. 1:**
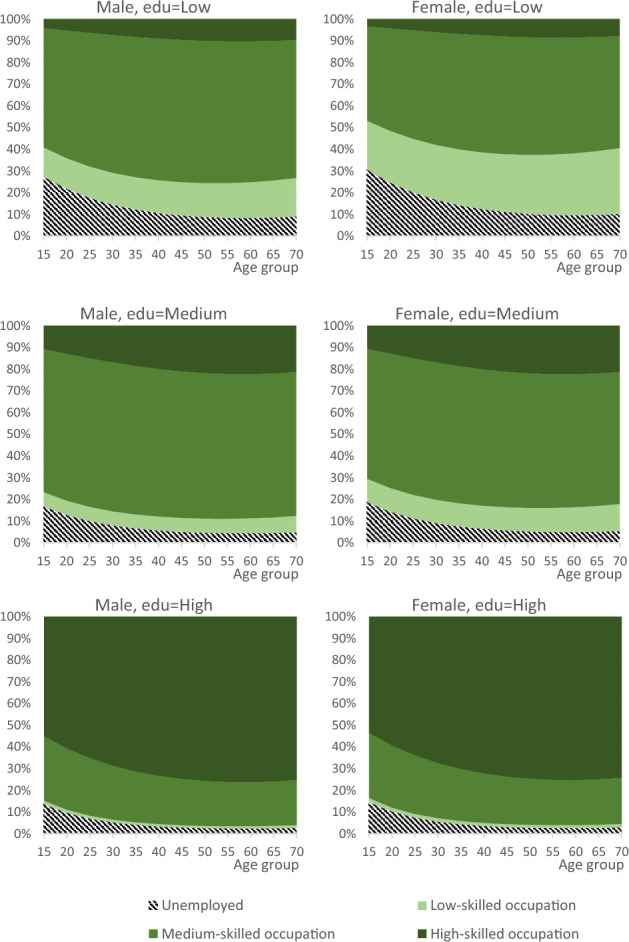
Predicted occupation of workers by age, sex and education

The specification contains three broad sets of covariates, which are linked by parameters that can be estimated to the probability of having a particular occupation. The first set of variables relates to *sociodemographic characteristics* such as age (AGEGR), sex (SEX), and education (EDU) (parameters ${\beta }_{1}$ to ${\beta }_{5}$). The estimated effects of these variables are summarized in Fig. 1, which presents the predicted occupational distribution for native-born individuals under the assumption that labour demand and supply equalize. The results highlight a strong relationship between education and occupation: individuals with higher educational attainment face a lower probability of unemployment and a higher probability of working in high-skilled occupations. Younger workers are more likely to be unemployed, whereas the probability of holding a high-skilled job increases with age, reflecting the accumulation of work experience. At each level of education, women are slightly more likely than men to be unemployed or employed in occupations below their qualification level.

The second set of covariates captures *immigrant-specific factors* including migrant status by region of birth, duration of stay, and age at immigration, together with their interactions with sex and education (linked to the outcome by parameters ${\beta }_{6}$ to ${\beta }_{8})$. The corresponding estimation results for the effect of these covariates are provided in the Supplementary Information (Fig. S1). Our results indicate that newly arrived immigrants have a lower probability of holding high-skilled jobs and a higher probability of being unemployed as compared to natives. The gaps narrow with time spent in the host country but remain large and converge more slowly for immigrants from Sub-Saharan Africa, West Asia, and North Africa, particularly among women. By contrast, immigrants who arrive during childhood (below age 15) have occupational distributions that closely resemble those of natives.

The third set of covariates relates to the *demand for labour* (linked to the outcome variable by the parameters ${\lambda }_{1}$ and ${\lambda }_{2}$). The model incorporates variables measuring country(*c*)-year(*t*)-specific labour demand by skill (*DemH* for high-skilled jobs, *DemM* for medium-skilled and *DemL* for low-skilled), normalized by the total labour force (LF). For estimation, labour demand for low- and medium-skilled jobs are combined to a single predictor, in order to improve stability in the estimated parameters and parsimony. Higher labour demand is found to reduce unemployment, while skill-specific demand shocks not only affect the likelihood of employment in the occupations that directly require this skill, but also generate spillover effects across other skill groups. A sensitivity analysis of these relationships is presented in the Supplementary Information (Fig. S2). In this robustness check, we compare the reference model (based on CEDEFOP demand estimates) with scenarios involving ± 25% changes in aggregate demand and ± 25% changes in the relative demand for high-skilled jobs. The results show that aggregate labour demand strongly affects unemployment rates, whereas changes in relative demand mainly alter the distribution of workers across occupations without significantly impacting unemployment.

The population projection used in our analysis begins in 2020 and is based on census data, which does not include information on occupation. We thus start by imputing the distribution of occupations for the 2020 base population using the multinomial model described above. We then calculate country and occupation-specific post-hoc adjustment factors that make the imputed distribution of workers by occupation match the actual distribution in 2020 and keep these calibration factors, which tend to be relatively small, constant throughout the projection period (see Table S2 in the Supplementary Information for the country-specific post-hoc adjustment factors). The adjusted probabilities ($\widehat{\text{Pr}})$ for country *c* and occupation *k* are therefore calculated as: $\widehat{\text{Pr}}\left({Y}_{i}=k\right|country=c)={\lambda }_{kc}\text{Pr}({Y}_{i}=k)/{\sum }_{j=1}^{K}{\lambda }_{jc}\text{Pr}({Y}_{i}=j)$.

The EU-LFS data for 2020 are affected by the labour market restrictions during the COVID pandemic lockdowns. Since the projection model is used to forecast long-term secular trends without taking into account such large period effects, we interpolate the occupation distribution for 2020 using data from 2014 to 2019 and of 2022, using linear interpolation.

### Projecting Labour Demand

In addition to the individual characteristics projected in the microsimulation model, the occupation module requires assumptions about the demand of jobs. First, we generate the labour supply by aggregating individuals in the labour force with high, medium and low educational attainment into the corresponding occupational skill level. Next, we formulate the assumptions about future demand in two steps. First, the country-specific total labour demand is calculated as a ratio of the population size and thus depends exclusively on demographic dynamics (see Table S3 in the Supplementary Information). Similar fixed ratio scenarios linking demand variables to population size has been applied in forecasting of healthcare demand (Bernini et al., 2024), for instance.

The assumption behind our approach is that changes in population affect the demand for goods and services, which in turn leads to changes in the demand for labour. According to theory, although labour demand is influenced by many other factors (market conditions, economic policies, etc.), in the short run the demand for consumption goods and services is its main driver, and consumption directly depends on population size (Mankiw, 2020). Taking CEDEFOP’s labour demand estimates for the period 2010–2019 and relating them to Eurostat’s population estimates, the ratio of labour demand to population shows a high degree of stability over time, although it can differ across economies (see Table S3 in the Supplementary Information). The standard deviation of this ratio within countries is below 0.05 for all countries, with average ratios that are generally between 0.4 and 0.6 (Luxembourg is the only outlier with a ratio of 0.77, due to the cross-border nature of its labour market, where a large share of jobs are filled by non-residents). Given this remarkable temporal stability in the observed data, it is reasonable in the reference scenario to assume that the ratio of labour demand to population remains constant until 2060 at the average level observed during the period 2010–2019 for each country. Although this ratio might change in the future, historical evidence suggests that drastic changes are unlikely within the projected time frame. To further assess the robustness of this assumption, we also perform a sensitivity analysis where the ratio is shifted upward or downward by one standard deviation (see the results in Table S4 of the Supplementary Information). The potential impact of broader structural changes is explored in the policy scenarios introduced in the second part of the analysis.

In the context of our modelling exercise, total demand is distributed across the three occupation skill groups (high-skilled jobs, medium-skilled jobs, and low-skilled jobs). We extrapolate the country-specific distribution of labour demand by occupation skill levels using CEDEFOP estimates from 2010 to 2019. To do so, we applied a generalized logit regression to the observed distribution of job demand by skill levels, with year and region dummies as predictors, which provided the parameters for extrapolating demand shares into the future. To validate the adequacy of the extrapolation, we examined residual plots from the generalized logit regression (Fig. S3 in Supplementary Information). The residuals are distributed evenly around zero without discernible temporal patterns, indicating that past dynamics are roughly consistent with a logistic trend which can be reasonably projected into the future as our reference scenario.

**Fig. 2:**
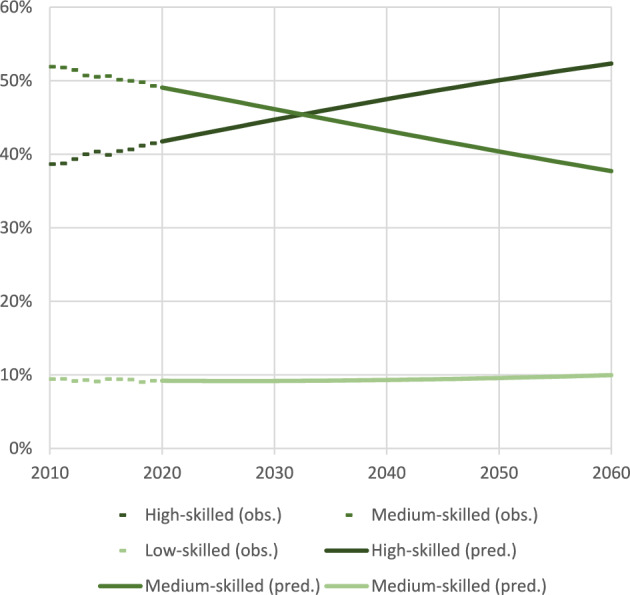
Distribution of labour demand by skill levels observed (2010–2019) and predicted (2020–2060), unweighted country average

Figure 2 presents the unweighted country average of the distribution of labour demand by skill levels projected to the year 2060. The extrapolated trends indicate a sharp increase in the share of demand for high-skilled workers, a corresponding decline for medium-skilled occupations, and a relatively stable demand for low-skilled jobs. These shifts reflect broader transformations in the economy (such as automation, digitalization, and the expansion of knowledge-intensive sectors) that are reshaping labour market needs. Together, they point to a deepening of occupational polarization, consistent with patterns identified in the literature (Acemoglu & Restrepo, 2019; Autor et al., 2013; Brynjolfsson & McAfee, 2014). In the microsimulation model, skill-specific demand is calculated by multiplying this distribution of labour demand by occupation by total labour demand.

## Results

### The Shift Towards High-Skilled Occupations and Greater Labour Mismatch

**Fig. 3:**
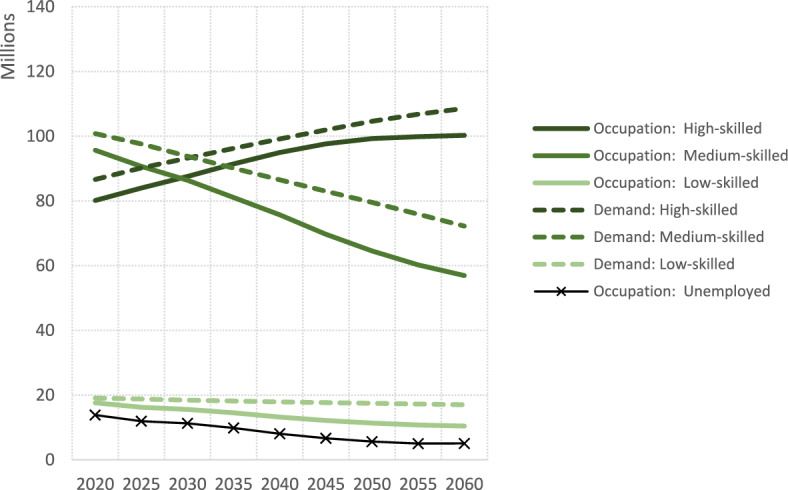
Projected workers and demand by occupation, European Union, 2020–2060

Having established the structure of the microsimulation model and its underlying assumptions, we now present projections of future occupational distributions and assess the extent of skill mismatch under the reference scenario. Figure 3 shows the projected trends in the number of available jobs and occupied jobs by skill up to the year 2060 for the EU. The gap between labour demand (dashed line) and jobs occupied (plain line) represents vacancies. Table 2 shows the figures for 2020 and 2060 together with the supply of workers by educational attainment and the corresponding percentage change.

**Table 2:** Changes in workforce (in millions) and labour demand (in millions) by education and occupation skill levels in the European Union, 2020 vs. 2060

	Low	Medium	High	Total
2020				
Workers by education	38.6	95.1	73.5	207.3
Workers by occupation	17.6	95.7	80.1	193.4
Labour demand	19.1	100.8	86.6	206.5
2060				
Workers by education	13.6	67.3	91.9	172.7
Workers by occupation	10.5	56.9	100.3	167.7
Labour demand	17.0	72.2	108.6	197.8
% Change				
Workers by education	− 65%	− 29%	25%	− 17%
Workers by occupation	− 41%	− 40%	25%	− 13%
Labour demand	− 11%	− 28%	25%	− 4%

Assuming that the total number of available jobs is a function of population size, future labour demand is expected to remain relatively stable as Europe’s population growth comes to an end. By 2060, our model predicts that labour demand in the EU will reach 198 million available jobs, compared to 207 million in 2020. However, when looking at the skill level of available jobs, a continuation of past trends results in a steep 25% increase in the demand for high-skilled workers, underscoring the growing need for a better skilled workforce capable of meeting the demands of an increasingly knowledge-based economy. Conversely, the total number of middle-skill jobs is projected to decline by nearly 30%, while the number of low-skill jobs will remain constant.

To assess the robustness of the assumption that labour demand is related to population size, we have also tested scenarios in which the relationship between was shifted one standard error upward or downward from the historical country-specific trend (see Table S4 in the Supplementary Information). This exercise serves to evaluate whether moderate deviations in the demand-population linkage such as those observed across European economies in the past decades, would alter our long-term projections. The results indicate that such variations have only a limited effect. By 2060, total labour demand would differ by about ± 6% compared to the reference, while the number of occupied workers would change by roughly 1%. The impact is somewhat more pronounced for high-skilled jobs, but the sensitivity-analysis confirms that the reference results are robust to plausible uncertainty in the demand-population ratio.

As a result of the continued expansion of higher education among younger cohorts, both the number and share of medium- and low-skilled workers are expected to decline rather faster than the number of jobs requiring such skills. Between 2020 and 2060, the combined supply of workers with low or medium levels of education is expected to decrease by around 40% (see Table 2 and Fig. S4), while labour demand for occupations requiring these skill levels is expected to decline by approximately 26%. These changes in the educational composition of the labour supply and demand are a key factor in determining future vacancy trends. Thus, while the vacancy rate for high-skilled jobs is expected to remain roughly constant (fluctuating between 4 and 7%, or between 4 and 8 million vacancies in absolute terms), the share of medium- and low-skilled job vacancies is projected to increase significantly. In 2020, 5% (5.1 million) and 8% (1.5 million) of medium- and low-skilled jobs, respectively, were unfilled. By 2060, these figures will reach 21% (15.3 million) and 39% (6.5 million), respectively. In summary, the total number of job vacancies is projected to more than double in 2060 compared to 2020 (30 million vs. 13 million). Of these, one fifth will require high skills, half will require medium skills, and the rest will require low skills.

**Fig. 4:**
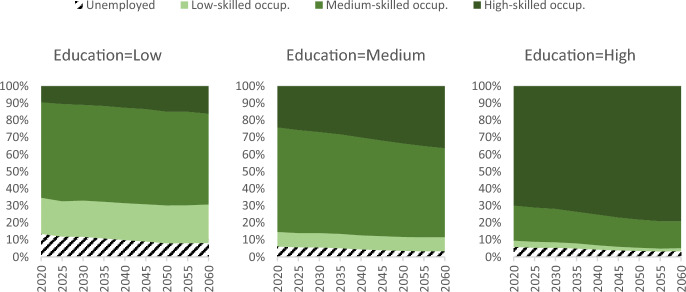
Projected occupational skill levels of workers by educational attainment, European Union, 2020–2060

From an economic perspective, however, the increasing number of job vacancies does not necessarily indicate a labour shortage. First, in addition to these vacancies, there are workers who cannot find jobs that match their skills. Although declining, this phenomenon is still significant in 2060, especially for highly educated workers (30% to 20% occupying less skilled job or unemployed, Fig. 4). In absolute terms, the projections imply that there will be 30 million workers (16%) in 2060 who are either overqualified or unemployed.

**Fig. 5:**
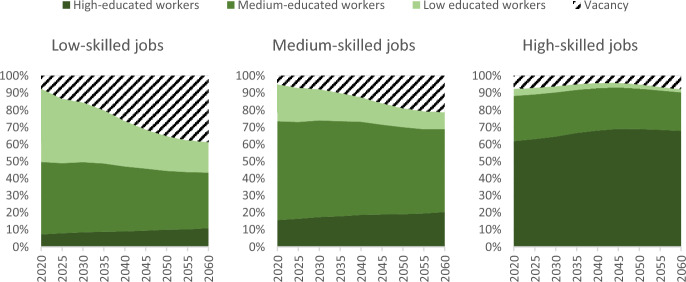
Projected distribution of jobs available according to the educational attainment of workers, European Union, 2020–2060

Second, a significant share of jobs will be filled by workers with lower skills (Fig. 5). In fact, throughout the projection, about a quarter of high-skilled jobs in 2060 will be occupied by workers with medium or low levels of education, with little change over time. This suggests that some mismatched workers with lower education than that corresponding to the skill level of the job they have tend to earn higher wages than their counterparts in matched jobs, and that their on-the-job skills and experience may compensate for their lower formal qualifications (Araújo & Carneiro, 2023). Supply constraints and rigid labour markets also affect skill mismatches as firms struggle to adjust skill composition of their workforce (Kampelmann et al., 2020).

The term “labour shortage” might not be an adequate concept to describe the situation, as it is not clear that the demand for labour exceeds the available supply of workers with the necessary skills (Pouliakas et al., 2024). This coexistence of vacancies and underutilization reflects a structural mismatch between the characteristics of jobs and those of available workers, rather than an aggregate labour shortage. Indeed, in addition to the availability of workers, other factors may explain the difficulty of filling a job. These include working conditions that do not meet workers’ expectations, a mismatch between the specific skills required and those available, geographic location mismatches, and too high expectations from employers in terms of criteria (Cappelli, 2015; Cascio & Boudreau, 2011; Mas & Pallais, 2017). As there is a discrepancy between the skills, qualifications, or preferences of workers and the requirements or conditions of available jobs, we label this growing number and share of high-skilled job vacancies as "labour mismatches" (Şahin et al., 2014).

### What-If Scenarios of Potential Policy Responses

Given the projected rise in job vacancies and underutilization under the reference scenario, we explore a series of policy scenarios designed to mitigate (some of) these labour market challenges. We design a set of policy scenarios based on three dimensions of response: immigration, human capital and employers. These what-if scenarios are designed with a dual objective: to reduce the job vacancy rate and to reduce the share of underutilized workers (unemployed or overqualified).

#### Immigration Response

Immigration is often seen as a response to labour needs (European Commission, 2024). Immigration policy in many countries is explicitly aimed at meeting the needs of the labour market. The modelling structure of *Link4Skills-Mic* incorporates heterogeneity in socioeconomic behaviour by place of birth across multiple dimensions, allowing for the construction of sophisticated policy scenarios. Here, we build two alternative scenarios related to immigration to the EU:

1. *High immigration scenario*. In this scenario, the total number of immigrants from the rest of the world towards the EU is increased by 50% from 2025, keeping every other exogenous variable constant. Since immigrants are more concentrated in working age groups than the native population, this can be seen as a simplistic "demographic" solution to labour market mismatch. This scenario thus assumes a drastically higher number of immigrants than Europe has traditionally received in the past, but still lower in proportion to that observed in Canada or Australia (Abel & Cohen, 2019), where immigration policy and numerical targets are explicitly designed in large part to respond to labour market needs.
2. *Better selection scenario.* There is evidence that selected immigrants tend to make a more positive contribution to the economy (Nannestad, 2007). This scenario maintains the same level of immigration as the reference scenario, but from 2025 onwards selects immigrants who are more likely both to participate in the labour force and to occupy medium- and high-skilled jobs. In practice, this scenario is implemented by imposing that all immigrants have the same educational attainment, labour force participation rates, and occupations as those from North America/Other Europe, which is the group with better outcomes in these areas. Since this scenario also affects labour force participation and occupation, one could rethink its narrative as relating to successful integration policies.

#### Human Capital Response

Education policies can help to better prepare the future workforce to meet the needs of the market (European Commission, 2020). We build three scenarios that aim at mimicking a better use of the increased human capital implied by increasing educational attainment.

3. *Better education scenario*. This scenario envisages a successful policy of improving access to post-secondary education for new cohorts. The odds of obtaining a secondary and post-secondary education are increased by 25% for cohorts that did not complete their education at the beginning of the projection (cohort born in 1990 and later). This assumption benefits countries that lag behind in educational attainment and the scenario leads to a narrowing of the gap in educational attainment across countries.
4. *Mid-career retraining scenario.* In this scenario, successful life-long learning programmes are implemented for workers with medium education, who acquire new and relevant skills. These new skills help them perform high-skilled jobs and remain competitive in the labour market in the face of changing industry demands. Specifically, this scenario assumes that in each 5-year increment starting in 2025, 5% of medium-skilled workers move from medium -skilled to high-skilled, and 5% move from low-skilled to medium -skilled. Over a 40-year career, this means that one-third of low- and medium-educated workers will move to the upper skill level at some point during their careers, which is roughly in line with OECD estimates (Nedelkoska & Quintini, 2018).
5. *Later retirement scenario.* Postponing retirement age is often seen as a solution to mitigate some of the consequences of population aging and to stimulate economic growth (see for example Kuhn & Prettner, 2023). In this scenario, older workers are incentivised to remain active and retire at older age, either by policies that increase minimum retirement age or introduce flexible transition to full retirement, resulting in longer engagement of older workers in the workforce. Starting in 2025, the labour force participation rate of the 50+ population is assumed to reach the level observed today in Sweden, which is among the highest in the world (OECD, 2024).

#### Employers’ Response

The two dimensions explored in the previous scenarios concern government responses to labour mismatch. In the two following scenarios, we concentrate on potential beneficial responses from the employer side.

6. *Automation scenario.* This scenario implies that employers invest in the widespread use of technology to perform routine tasks or processes with minimal human intervention. The OECD estimates that about 14% of jobs are highly automatable, while another 32% have many tasks that could be automated (Nedelkoska & Quintini, 2018). This process can in principle be applied to all types of jobs, for example via the use of artificial intelligence or software automation for high-skilled jobs, mechanical automation for medium-skilled jobs, or manual automation for low-skilled jobs. In this scenario, we assume that the overall labour demand will be gradually reduced by 15% by 2060 in comparison to the reference scenario.
7. *Upskilling scenario.* Employers invest in internal training programs to upskill their employees. To come to terms with high competition for specific skills, employers lower their hiring requirements and are open to hire workers for their potential and develop their skills internally. Our assumption implies that from 2025 onwards, workers are 25% more likely (in terms of odds ratio) to be hired for a job that requires higher skills, as it is assumed that employers will hire less skilled workers and compensate for the lack of skills through in-house training.

**Fig. 6:**
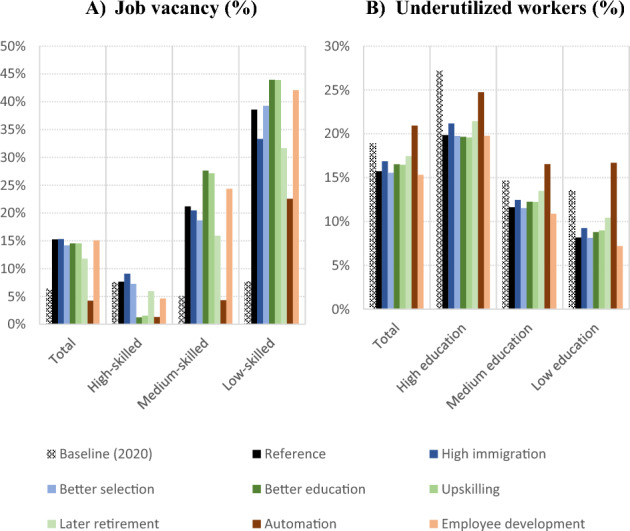
Job vacancy rate by skills required and proportion of underutilized workers by educational attainment, European Union, 2020 (baseline) and 2060 (all scenarios)

Figure 6 shows the job vacancy rate by required skill and the share of underutilized (overqualified or unemployed) workers by educational attainment for 2060 in these six policy scenarios, as compared to the reference scenario. Detailed results are shown in Table S5 in the Supplementary Information.

The immigration response scenarios have very little quantitative impact on the labour market overall. For the scenario with increased international immigration, the only notable impact is a slight reduction in the share of low-skilled job vacancies (33% vs. 39%), due to the higher propensity of immigrants to work in the low-skilled job segment. Given that labour markets in 2060 are assumed to be dominated by high-skilled jobs, this slight reduction does not have a significant impact on the overall rate of job vacancies. While higher immigration increases the number of workers, it also increases the demand for workers through its effect on consumption via population size preventing the system from reaching equilibrium. In reality, such feedback effects may stabilize through economic or institutional constraints. Although immigrants from outside the EU are more concentrated in the working-age population, they are also less likely to work during the first years after their arrival and less likely to occupy high-skilled jobs as compared to the EU-born population, resulting in a marginal impact on the overall vacancy rate in 2060 and on the share of workers who are overqualified or unemployed. This finding is consistent with the economic literature, which shows that immigration (in the intensity observed hitherto) has a small impact on aggregate labour market outcomes, and when it does, it tends to affect low-skilled workers (Longhi et al., 2010).

For similar reasons, the projection results of the *Better Selection* scenario are not much different from the reference scenario. Although immigrants are more educated and more likely to work in comparison to the reference scenario, their numbers are not as high as in the *High Immigration* scenario and therefore do not significantly affect national trends.

The human capital response to the labour market challenges leads to different labour market dynamics. The calibration of scenarios that contain narratives based in increasing the educational attainment of younger cohorts (*Better Education*) as well as the skills of the workforce (*Mid-career Retraining*) lead to employment dynamics that are able to fill the required number of high-skilled jobs by 2060. By shifting workers from lower to higher educational groups, these scenarios increase the rate of medium- and low-skilled job vacancies and, in sum, the total rate of job vacancies in 2060 remains similar to that projected in the reference scenario (15%). The scenario with increased participation of older people is the only one that slightly reduces the overall job vacancy rate (12% vs. 15%), while having no notable impact on the proportion of overqualified or unemployed individuals. Nevertheless, the job vacancy rate is still twice as high as in 2020 (6%).

The *Upskilling* scenario results in a slight decrease of the vacancy rate for high-skilled jobs (5% vs 8%), together with a higher rate for medium-skilled (24% vs. 21%) and low-skilled (42% vs. 39%) jobs, but no significant effect on the overall vacancy rate and a small but positive effect on the share of underutilized workers. The *Automation* scenario is the only one that leads to a drastic reduction in the overall vacancy rate. By 2060, a 15% reduction in labour demand due to automation would reduce the job vacancy rate to 4%, compared to 15% in the reference scenario. Under this scenario, the vacancy rate is particularly reduced for high-skilled jobs (1% vs. 8%), which are expected to dominate the labour market by the end of the projection horizon. This scenario substantially increases the proportion of underutilized workers.

## Discussion and Conclusion

The projections presented in this study offer a nuanced view of future labour market dynamics in the EU. Beyond presenting novel empirical results, this study makes a methodological contribution by integrating demographic, educational, and labour market processes within a single microsimulation framework. Unlike traditional macro-level or econometric forecasting models, Link4Skills-Mic represents the joint evolution of individuals’ demographic and socio-economic trajectories, allowing interactions and feedback effects between modules to emerge endogenously. This design makes it possible to examine how educational expansion, ageing, and migration simultaneously influence labour supply, occupational allocation, and mismatch dynamics over time. It also enables the assessment of policy interventions across multiple dimensions such as demographic change, human capital, and employer responses within a consistent analytical setting. By capturing these interdependencies, the model reveals that projected imbalances are not simply driven by declining labour supply but by the structural misalignment between demographic composition, educational attainment, and evolving job demand, an insight that simpler projection frameworks cannot provide.

While demographic and educational trends are expected to expand the supply of high-skilled workers, this development alone appears unable to eliminate labour market mismatches. Available skill projection exercises highlight increasing demand for highly-skilled workers, particularly in knowledge-intensive industries such as information technology, healthcare, and professional services, e.g. legal, social, and business-related professions (CEDEFOP, 2023). Countries such as Germany and the Netherlands report acute shortages of engineers, IT specialists, and healthcare professionals (CEDEFOP, 2023). This aligns with trends observed in the U.S. labour market, where the BLS projects that healthcare and technology sectors will generate the most job opportunities over the next decade (Bureau of Labor Statistics, 2024). Our projection exercises indicate that while the supply of high-skilled workers will expand substantially, the demand for high-skilled labour is projected to grow even faster, resulting in a persistent increase in unfilled high-skilled vacancies by 2060. It is important to note that the projected increase in job vacancies does not necessarily imply an aggregate shortage of labour. Our results highlight the problem of underutilization in the labour market, reflecting persistent skill and expectation mismatches in the labour market rather than gross labour shortages. This coexistence of surplus labour and persistent vacancies highlights that the underlying problem is one of structural mismatch between skills, job characteristics, and hiring practices, rather than a quantitative insufficiency of workers. This insight extends those gained by existing projections by CEDEFOP, the U.S. Bureau of Labor Statistics, and the World Economic Forum, which consistently identify labour market mismatches as a key labour market challenge (Bureau of Labor Statistics, 2024; CEDEFOP, 2023; WEF, 2025). These mismatches occur across multiple dimensions, including geography, skill sets, and sectoral needs. For example, CEDEFOP’s analysis underscores that while some regions in Europe experience labour shortages, others continue to struggle with high unemployment rates, particularly among young workers. Employers in advanced economies frequently report difficulties in recruiting professionals with expertise in artificial intelligence, data science, and cybersecurity, highlighting the disconnect between educational outputs and labour market needs (CEDEFOP, 2023; WEF, 2025). However, poor wages and unattractive working conditions are often understood as root causes of labour shortages (Pouliakas et al., 2024), as well as lack of on job training and inefficient hiring practices (Feist, 2024).

We present several scenarios showcasing potential policy responses to these labour market challenges, some of which are highlighted in existing strategies and policy documents. The European Commission (2020) and CEDEFOP (2019) have warned of a widening skills gap, exacerbated by slow adaptation of educational systems and insufficient investment in lifelong learning programs. Three of our scenarios—*Better education, Mid-career retraining* and *Upskilling* illustrate potential effects of successful implementation of education and on-job training reforms.

The goals of reducing the vacancy rate and the percentage of overqualified or unemployed workers are often in conflict. Greater competition among workers for available jobs typically leads to fewer vacancies, but results in a higher proportion of workers unable to find jobs that match their skills (Blanchard et al., 1989; Pissarides, 2000). Solutions based on migration responses appear to have very little impact due to its counteracting effect on both labour supply and demand. Taken alone, human capital solutions also lead to balancing effects on vacancies. The scenario of later retirement is the only one that, on its own, reduces the overall vacancy rate without drastically increasing the share of underutilized workers. However, this policy response alone is not sufficient to reverse the broad trends in the labour market and a combination of multiple measures would be needed to simultaneously reduce mismatches and worker underutilisation.

From the perspective of employers, reaping the benefits of automation to reduce their demand for workers appears as the most successful approach in our simulations. Reducing the need for workers by just 15% by 2060 through robotics, artificial intelligence, and other automation processes could substantially reduce job vacancy rates, especially for high-skilled jobs. From the perspective of workers, however, this solution is likely to increase underutilization. Indeed, existing studies suggest that automation can lead to job losses and sectoral shifts (Acemoglu & Restrepo, 2018). At the same time, it can also create new types of higher-quality jobs required by automation technologies and increase overall productivity and economic efficiency through lower production costs (Vermeulen et al., 2018). For these reasons, the implementation of proactive responses that prioritize reskilling and upskilling of workers and which address the negative effects of automation on unemployment, especially for vulnerable groups, is essential in this context. Effective public policies to address labour mismatches require collaboration across sectors and across levels of governance. Our results indicate a need for a holistic approach, integrating education, training and employment strategies into a comprehensive plan to effectively address labour market mismatches.

Our model has several limitations that should be noted. First, the distribution of occupations is heavily influenced by our labour demand assumptions. For the reference scenario, we assume that overall labour demand moves hand in hand with population size and that increasing trends in the demand for high-skilled jobs are expected to continue. The model does not incorporate macroeconomic feedback mechanisms and abstracts from the fact that labour demand is influenced by a variety of factors, including economic conditions, economic cycles, government policies, technological advances, and industry trends (Blanchard & Johnson, 2012). Projecting the future trajectory of these factors is inherently challenging. In addition, our model does not fully account for the dynamic interplay between changes in labour supply and demand over time, as these factors can interact in complex ways and affect labour demand across sectors and regions via the effect of such interactions on wages.

Second, the model categorizes job requirements and education into broad skill levels (low, medium, high), thus hiding important nuances within these categories. This is particularly true for high-skilled and medium-skilled jobs, which encompass a range of highly specialized fields that are not easily interchangeable. This simplification affects the accuracy of matching workers to jobs, as the process involves more factors than just the general skill level.

Third, because the microsimulation relies on demographic projections, it shares the common limitations of other population projection models. These models depend on assumptions about future fertility, mortality, and migration, and the uncertainty in these projections increases as the time horizon lengthens (Keyfitz, 1981). Importantly, characteristics-based microsimulation modelling is based on a one-to-one correspondence between a worker and a job. This simplification is necessary within our modelling framework, however, in real economy one worker can occupy more than one vacancy (i.e. working part-time for multiple employers). In addition, job vacancies can also be occupied by cross-border workers who are residents in a country but work in a different one. Our modelling is based on residency criterion; hence, we assume that country-specific labour demand is filled by residents (native and immigrants) of the country.

Finally, the microsimulation of our model produces deterministic scenario-based results rather than probabilistic forecasts, and it therefore does not provide formal confidence intervals around the projections. Uncertainty is addressed indirectly through sensitivity analyses and by entertaining alternative policy or demographic scenarios, which illustrate the potential range of outcomes without quantifying their statistical likelihood. This limitation is inherent to long-term projection exercises of this type and should be borne in mind when interpreting the results.

## Supplementary Information

Below is the link to the electronic supplementary material.

Supplementary file1 (DOCX 129 KB)

## Data Availability

Code and parameter files for the Reference scenario, along with detailed projection outcomes for all scenarios, are available on Zenodo at https://zenodo.org/uploads/17191028. Parameter sets and code for other scenarios, and details on parameter calculations, are available from the corresponding author.

## Footnotes

1 While QuantMig-Mic was implemented in ModGen, the present model is coded in SAS following the operative microsimulation structure proposed by Marois and KC (2021)..

2 Labor demand for a given occupation is estimated by summing employment in that occupation and job openings. Job openings refer to positions within organizations that are currently vacant and available for potential candidates to apply for, including new positions and vacancies resulting from departures.
