# Supplementary material for: Projecting Labour Market Imbalances and Skill Mismatch Under Demographic Change in the EU
Source: Eur J Popul. 2025 Dec 4;42(1):4. doi: 10.1007/s10680-025-09758-2 (PMC12789361; doi:10.1007/s10680-025-09758-2)
Supplement: Supplementary file 1 — Supplementary file1 (DOCX 129 KB) [file 10680_2025_9758_MOESM1_ESM.docx]

## Projecting Labour Market Imbalances and Skill Mismatch under Demographic Change in the EU

## Supplementary Information (SI)

**Authors:**

*Guillaume Marois^1,2^

Michaela Potančoková^1^

Agnieszka Bezat^3^

Jesús Crespo Cuaresma^4,1^

1. Wittgenstein Centre for Demography and Global Human Capital (IIASA, VID/OeAW, University of Vienna)
2. Asian Demographic Research Institute, School of Sociology and Political Sciences, Shanghai University, Shanghai, China
3. Department of Quantitative Methods and Information Technology, Kozminski University
4. Department of Economics, Vienna University of Economics and Business (WU), Vienna, Austria

*Corresponding author: marois@iiasa.ac.at

## S1 - Link4skills microsimulation model

### Overview

*Link4Skills-Mic* is a dynamic microsimulation model specifically designed to project labour supply and demand by occupational skill group in the EU27. While its structure and modelling of core demographic events are inspired by earlier microsimulation frameworks (CEPAM-Mic or QuantMig-Mic, Marois et al., 2023), it is implemented in SAS rather than in ModGen. This choice of platform is made to allow greater flexibility in including feedback mechanisms between labour supply and labour demand, a necessary feature when modelling the dimension of skill mismatch. In our approach, labour demand is treated as an aggregated country-level variable derived as a function of population size, while individuals’ occupational outcomes are modelled at the micro level. Implementing the model in SAS facilitates the integration of such aggregate-to-individual linkages, enabling the simulation of interactions between macro-level demand and individual labour supply dynamics.

The model tracks individuals along multiple dimensions, including age, sex, country of residence, place of birth, educational attainment, parental education, labour force status, employment status, and occupation. Migration-related characteristics such as age at immigration and duration of residence are also included, ensuring that heterogeneity in demographic and labour market behaviours is explicitly represented. *Link4Skills-Mic* operates in discrete time, following the approach outlined by Marois and KC (2021). The simulation advances in 5-year steps. At each step, demographic and socio-economic events (fertility, mortality, migration, educational transitions, labour force participation, occupational allocation) are probabilistically assigned to individuals based on hazard models or transition probabilities.

The synthetic microdata set is derived from census aggregates. We start from population counts based on the 2011 census and convert them into 3.4 million individual records that are representative by age, sex, country, education, and migration background. Such a sample size balances computational feasibility with analytical precision and ensures robust coverage of relevant population subgroups. We run a pre-simulation that reproduces observed events over the period 2011–2019 (births, deaths, educational progression, international and intra-EU migration, and labour force transitions) to obtain a starting population for the year 2020 that aligns with the observed demographic and socio-economic population structures.

### From this baseline, the model projects forward annually until 2060. At each step, individual characteristics are updated using probabilistic rules for education progression, intra-EU mobility, fertility, mortality, and international migration. Births and immigrants add new cases, while deaths and extra-EU emigrants are removed from the population which is tracked. Labour force participation is overlaid on the population counts: for each individual, labour force status is assigned probabilistically as a function of their characteristics, without altering the underlying population totals. Among those classified as active, an occupation is allocated based on their attributes and the skill-specific labour demand. This overlay allows us to compute mismatches between projected supply and demand across skill groups.

### The following sections summarize the main demographic and education modules of the model, together with their underlying assumptions. The occupation module, which is novel to this paper, is described in detail in the main manuscript. Projection code and detailed parameter values for the Reference scenario are available in the online repository, along with detailed projection outcomes for all scenarios (<https://zenodo.org/uploads/17191028>).

### Fertility module

The fertility module generates births during the simulation and introduces newborn individuals into the population. Births are simulated using age- and education-specific fertility rates at the country level, calibrated to match the 2023 update of the Wittgenstein Centre’s Shared Socioeconomic Pathways (SSP2) scenario (KC et al., 2024). In this scenario, future fertility trajectories are based on updated baseline estimates, with long-term values determined by expert opinion and intermediate values obtained through interpolation. The SSP2 scenario assumes a slight rebound of fertility in most European countries, with the total fertility rate in the EU increasing from around 1.5 children per woman in 2020–2024 to about 1.6 by the mid-2050s. It also incorporates recent trends, including unexpected fertility declines in Northern Europe and France, as well as short-term disruptions linked to the COVID-19 pandemic.

To incorporate heterogeneity by migration status, the rates are adjusted using odds ratios estimated from the EU labour force survey (EU-LFS), which captures fertility differentials by mother’s place of birth and duration of residence, while controlling for age and education (Potančoková & Marois, 2020). This results in systematically higher fertility for recently arrived immigrants from Africa and the Middle East, with the fertility gap declining but not fully closing as the duration of stay increases.

In the model architecture, when a birth occurs, a new individual is created with demographic and socio-economic characteristics assigned at birth. These include sex (drawn randomly to match natural sex ratios), country of residence, and intergenerational transmission of education. The mother’s education influences the child’s eventual educational attainment through the logic implied by the education module. The newborn then enters the simulation and is subject to the same probabilistic life course events as the rest of the population.

### Mortality module

The mortality module removes individuals from the simulated population through death events. Survival ratios are defined by age, sex, country of residence, and educational attainment, and are calibrated making use of the parameters from the 2023 update of the Wittgenstein Centre’s SSP2 scenario (KC et al., 2024). In this scenario, life expectancy continues to rise throughout the projection horizon, although the impact of the COVID-19 pandemic on mortality in the early 2020s is included.

Long-term assumptions are based on expert opinion regarding the pace of mortality decline and convergence across countries, with intermediate values obtained through interpolation. Life expectancy at birth is projected to exceed 90 years in most European countries by 2060, while differences by sex and country decrease steadily over time. Educational differentials are maintained: individuals with high education live longer than those with low education, with gaps averaging about six years for men and four years for women at age 15. Deaths are generated stochastically at the individual level. Once a death event occurs, the individual is removed from the simulated population.

### Education module

The education module follows the approach developed by Marois et al. (2019), updated with the most recent data. It assigns the highest level of education that each individual will eventually attain, while simulating progression through the schooling system. Educational attainment is classified into three levels (low, medium and high) following ISCED 2011. Probabilities of reaching each level are estimated using an ordered logit model applied to European Social Survey data, with predictors that include sex, birth cohort, mother’s education, and region of birth. This method captures intergenerational transmission, as well as sociocultural differentials in education. Country-specific cohort parameters are extrapolated to future years to reflect the continuation of the educational expansion. Long-term assumptions are consistent with the Wittgenstein Centre’s SSP2 scenario, which assumes convergence toward high levels of post-secondary attainment in most EU countries. In the reference scenario, the share of young adults (aged 30–34) with low education is projected to decline from 12.9% in 2020 to 7.7% in 2060, while the share of those with medium education decreases from 47.9% to 39.8%. Conversely, the proportion with high education increases from 39.2% in 2020 to 52.5% in 2060.

At birth (or upon immigration during childhood), each individual is probabilistically assigned their ultimate educational level. Schooling is then simulated dynamically: individuals enter education at age 5 and progress until completing their assigned level or reaching age 29. This allows the model to distinguish between current level of schooling and the highest educational level attained.

Education influences several other modules, including fertility, mortality, labour force participation, and occupational allocation.

### Migration

The migration module simulates population movements both within the EU (intra-EU mobility) and between the EU and the rest of the world (immigration and emigration). It follows the framework laid down in the QuantMig-Mic tool (Marois et al., 2023), and is implemented without modification in *Link4Skills-Mic*.

- Intra-EU mobility: Individuals can move between EU countries based on age-, sex-, education-, and origin-specific probabilities derived from the EU-LFS and calibrated to median bilateral migration flows (Aristotelous et al., 2022).
- International immigration: Immigrant inflows are disaggregated by eight broad regions of birth: Other Europe (e.g. Turkey, Ukraine, Western Balkans), North Africa, Sub-Saharan Africa, West Asia, South & South-East Asia, East Asia, Latin America, and North America & Oceania. The number of new arrivals is based on the average annual migration flows observed between 2011 and 2019. Specifically, average emigration rates from each world region are calculated and applied to the projected population size of the 20–34 age group in each region. This procedure results in projected flows that grow over time from demographically young regions (e.g. Sub-Saharan Africa, West Asia) and decline from ageing regions (e.g. East Asia, Other Europe). For example, under the baseline assumption, the EU is projected to receive about 15.3 million immigrants during 2020-2024, falling to 12.7 million in 2035-2039 and 12.4 million in 2055-2059. The socio-demographic characteristics of immigrants (sex, age at arrival, education) are assigned using distributions from the EU-LFS for recent arrivals (0-4 years of residence).
- International emigration: Individuals can leave the EU permanently based on emigration rates by country of residence and place of birth, also derived from QuantMig estimates. In the baseline assumption, annual emigration flows amount to around 7.3 million in the period 2020-2024, decreasing slightly to 6.8-6.9 million per 5-year period by mid-century. Once emigrated, individuals are removed from the simulation and no longer contribute to demographic or labour force outcomes.

This module captures heterogeneity in migration dynamics, particularly differences by origin region, age at arrival, and duration of residence, which also influence fertility and labour force participation outcomes in the model.

### Labour force participation module

The labour force participation module determines whether individuals are in or out of the labour force at each step of the simulation. This module and its parameters correspond to those developed in QuantMig-Mic (Marois et al., 2023), ensuring consistency with earlier population projections that explicitly model immigrant integration. The base probabilities of labour force participation are estimated using the cohort-development approach, where age-, sex-, and education-specific participation rates are calculated by cohort for each country by reconstructing working life tables from cross-sectional entry and exit rates. This method ensures that older women will participate more in the future in countries that already have relatively high labour market participation rates for young women. Odds ratios estimated from logistic regression are applied to these participation rates, capturing differentials by place of birth, duration of stay, and age at immigration. All estimates are derived from EU-LFS data for the years 2014-2019, which provide sufficiently long pre-COVID trends to avoid distortions from short-term shocks.

Baseline participation rates in 2020 were 38.6% for ages 15–24, 85.3% for ages 25–54, and 36.3% for ages 55–74. These are projected to change slightly by 2060 to 36.2%, 85.5%, and 42.5%, respectively, reflecting stable prime-age participation but gradual increases among older workers. Immigrant-specific effects are included: controlling for education and age, immigrants from some regions (notably North Africa and the Middle East) have systematically lower participation rates, especially among women and recent arrivals (0–4 years of residence), as shown in Table S1. These gaps narrow with duration of stay but remain significant, reflecting persistent barriers such as linguistic difficulties, non-recognition of qualifications, and weaker professional networks. The labour force status estimated in this module conditions the occupation allocation module, so only those in the labour force are subsequently distributed into employment or unemployment by occupational skill group.

**Table S1: Parameters for the immigration-related variables used in the regression model predicting the labour force participation (control for age, sex, education, and country of residence)**

| **Age at immigration** | **Place of birth** | **Duration of stay (number of years)** | **Males** | | **Females** | |
| --- | --- | --- | --- | --- | --- | --- |
| NA | Born in EU+ | NA | Ref |  | Ref |  |
| <15 | Born outside EU+ | All | -0.086 | *** | -0.289 | *** |
| >=15 | Born in another European country / North America / Oceania | 0-4 | -0.544 | *** | -1.554 | *** |
|  |  | 5-9 | -0.271 | ** | -1.268 | *** |
|  |  | 10+ | -0.485 | *** | -0.969 | *** |
|  | Born in North Africa / Middle East | 0-4 | -1.85 | *** | -2.806 | *** |
|  |  | 5-9 | -0.872 | *** | -2.188 | *** |
|  |  | 10+ | -0.585 | *** | -1.446 | *** |
|  | Born in Other Africa | 0-4 | -1.116 | *** | -1.516 | *** |
|  |  | 5-9 | -0.585 | *** | -1.072 | *** |
|  |  | 10+ | -0.178 | ** | -0.515 | *** |
|  | Born in Latin America | 0-4 | -0.91 | *** | -1.396 | *** |
|  |  | 5-9 | -0.179 |  | -0.771 | *** |
|  |  | 10+ | -0.045 |  | -0.403 | *** |
|  | Born in Other Asia | 0-4 | -1.398 | *** | -2.242 | *** |
|  |  | 5-9 | -0.383 | *** | -1.665 | *** |
|  |  | 10+ | -0.061 |  | -1.017 | *** |
| *** p<0.0001 ; **p<0.01 | | | | | | |

Source: Marois, Potančoková, and González-Leonardo (2023).

## S2 – Additional Tables and Figures

**Table S2. Model fit statistics for the multinomial logit model used in Equation (1)**

| **Criterion** | **Intercept Only** | **Intercept and Covariates** |
| --- | --- | --- |
| **AIC** | 2,640,263 | 2,171,680 |
| **SC** | 2,640,299 | 2,174,515 |
| **-2 Log L** | 2,640,257 | 2,171,206 |

**Figure S1. Predicted occupation of workers by place of birth, duration of stay and age at immigration from equation 1.**

|  |
| --- |
|  |
|  |
|  |
|  |
|  |
|  |
| *For age group=30-34, demand = supply in each skill category. |

**Figure S2. Predicted occupation of workers by education from equation 1 according to different labour demand assumptions**

**Table S2. Post-hoc adjustment factors by country**

| Country | High-skilled occupation | Medium-skilled occupation | Low-skilled occupation | Unemployment |
| --- | --- | --- | --- | --- |
| AT | 1.15 | 1.00 | 0.91 | 0.50 |
| BE | 0.96 | 1.05 | 1.28 | 0.74 |
| BG | 0.85 | 1.00 | 1.62 | 1.28 |
| CY | 0.90 | 1.00 | 1.68 | 0.84 |
| CZ | 1.12 | 1.02 | 0.77 | 0.38 |
| DE | 1.02 | 1.02 | 1.01 | 0.63 |
| DK | 1.15 | 0.87 | 1.14 | 0.74 |
| EE | 0.91 | 1.09 | 1.27 | 0.84 |
| ES | 0.99 | 1.09 | 1.12 | 0.75 |
| FI | 1.03 | 1.00 | 0.79 | 1.03 |
| FR | 1.08 | 0.95 | 1.06 | 0.85 |
| GR | 0.75 | 1.05 | 0.72 | 2.10 |
| HR | 1.03 | 0.97 | 0.94 | 1.18 |
| HU | 0.97 | 0.99 | 1.27 | 0.89 |
| IE | 0.97 | 1.05 | 0.98 | 0.93 |
| IT | 1.07 | 0.94 | 1.04 | 1.07 |
| LT | 0.79 | 1.14 | 1.84 | 1.47 |
| LU | 1.12 | 0.84 | 0.78 | 1.33 |
| LV | 0.93 | 1.00 | 1.78 | 0.74 |
| MT | 1.22 | 0.89 | 0.72 | 1.32 |
| NL | 1.12 | 0.95 | 0.90 | 0.59 |
| PL | 1.07 | 1.07 | 0.84 | 0.40 |
| PT | 1.37 | 1.00 | 0.67 | 0.57 |
| RO | 0.95 | 1.05 | 1.05 | 0.68 |
| SE | 1.26 | 0.95 | 0.53 | 0.58 |
| SI | 1.08 | 0.93 | 0.95 | 1.07 |
| SK | 1.18 | 1.06 | 0.92 | 0.44 |

**Table S3. Ratio of total labour demand to population size, average of 2010-2019**

| Country | Average | S.D. |
| --- | --- | --- |
| AT | 0.52 | (0.01) |
| BE | 0.43 | (0.01) |
| BG | 0.51 | (0.03) |
| CY | 0.48 | (0.04) |
| CZ | 0.51 | (0.02) |
| DE | 0.55 | (0.01) |
| DK | 0.52 | (0.01) |
| EE | 0.49 | (0.03) |
| ES | 0.42 | (0.03) |
| FI | 0.49 | (0.01) |
| FR | 0.43 | (0.00) |
| GR | 0.42 | (0.03) |
| HR | 0.40 | (0.03) |
| HU | 0.45 | (0.04) |
| IE | 0.45 | (0.03) |
| IT | 0.43 | (0.01) |
| LT | 0.47 | (0.04) |
| LU | 0.77 | (0.02) |
| LV | 0.46 | (0.03) |
| MT | 0.48 | (0.04) |
| NL | 0.56 | (0.02) |
| PL | 0.43 | (0.01) |
| PT | 0.47 | (0.03) |
| RO | 0.44 | (0.01) |
| SE | 0.52 | (0.01) |
| SI | 0.48 | (0.03) |
| SK | 0.44 | (0.02) |

**Figure S3. Residual plot from the logit regression of job demand shares by skill levels, year, and region.** Residuals are defined as the difference between observed and predicted job demand shares


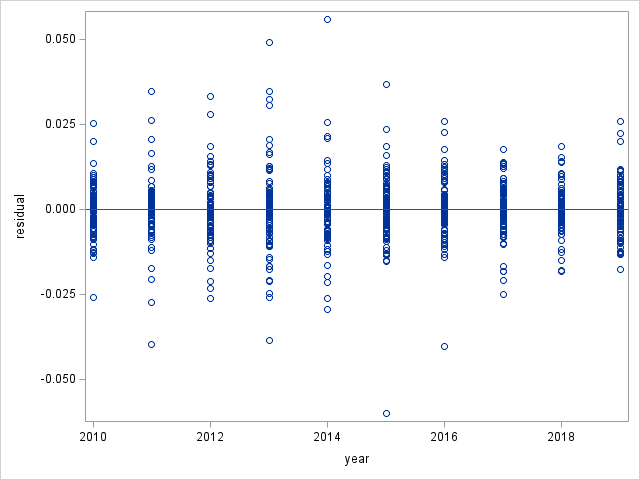


**Figure S4. Projected labour force by educational attainment, European Union, 2020-2060**

**Table S4. Sensitivity analysis of projected labour demand and occupational distribution of workers in the EU27 (2020–2060) to assumptions linking job demand to population size**

|  | **Skill group** | **2020** | **2060** | | |
| --- | --- | --- | --- | --- | --- |
|  |  |  | *Central* | *-1 S.D. labour demand/population* | *+1 S.D. labour demand/population* |
| **Workers by  occupation (in million)** | *Low* | 17.6 | 10.5 | 10.7 | 10.3 |
|  | *Medium* | 95.7 | 56.9 | 56.8 | 56.1 |
|  | *High* | 80.1 | 100.3 | 98.7 | 102.1 |
|  | *Total* | 193.4 | 167.7 | 166.2 | 168.4 |
| **Job demand (in million)** | *Low* | 19.1 | 17.0 | 16.4 | 17.6 |
|  | *Medium* | 100.8 | 72.2 | 69.9 | 74.5 |
|  | *High* | 86.6 | 108.6 | 105.9 | 111.2 |
|  | *Total* | 206.5 | 197.8 | 192.2 | 203.3 |

**Table S5. Projected job vacancy rate by skills required and proportion of underutilized**

**workers by educational attainment, European Union, 2040 and 2060**

| Scenario | | Job vacancy (%) | | | | Underutilized workers (%) | | | |
| --- | --- | --- | --- | --- | --- | --- | --- | --- | --- |
|  |  | *Total* | *High-skilled* | *Medium-skilled* | *Low-skilled* | *Total* | *High education* | *Medium education* | *Low education* |
| *2020* | | 6% | 7% | 5% | 8% | 19% | 27% | 15% | 14% |
| 2040 | *Reference* | 10% | 4% | 13% | 26% | 17% | 23% | 13% | 10% |
|  | *High immigration* | 10% | 5% | 13% | 23% | 18% | 24% | 13% | 10% |
|  | *Better selection* | 10% | 4% | 12% | 27% | 17% | 23% | 13% | 10% |
|  | *Better education* | 9% | 0% | 15% | 31% | 18% | 23% | 13% | 10% |
|  | *Mid-career retraining* | 9% | 0% | 15% | 31% | 18% | 23% | 13% | 10% |
|  | *Later retirement* | 6% | 4% | 7% | 16% | 20% | 26% | 15% | 13% |
|  | *Automation* | 4% | 1% | 5% | 17% | 19% | 25% | 15% | 13% |
|  | *Upskilling* | 10% | 1% | 15% | 32% | 17% | 23% | 12% | 8% |
| 2060 | *Reference* | 15% | 8% | 21% | 39% | 16% | 20% | 12% | 8% |
|  | *High immigration* | 15% | 9% | 20% | 33% | 17% | 21% | 12% | 9% |
|  | *Better selection* | 14% | 7% | 19% | 39% | 16% | 20% | 12% | 8% |
|  | *Better education* | 15% | 1% | 28% | 44% | 17% | 20% | 12% | 9% |
|  | *Mid-career retraining* | 15% | 2% | 27% | 44% | 16% | 20% | 12% | 9% |
|  | *Later retirement* | 12% | 6% | 16% | 32% | 17% | 21% | 13% | 10% |
|  | *Automation* | 4% | 1% | 4% | 23% | 21% | 25% | 17% | 17% |
|  | *Upskilling* | 15% | 5% | 24% | 42% | 15% | 20% | 11% | 7% |

## References

Aristotelous, G., Smith, P. W. F., & Bijak, J. (2022). *Technical report: Estimation methodology* (QuantMig Deliverable 6.3). University of Southampton.

KC, S., Dhakad, M., Potančoková, M., Adhikari, S., Yildiz, D., Mamolo, M., Sobotka, T., Zeman, K., Abel, G., Lutz, W., & Goujon, A. (2024). *Updating the Shared Socioeconomic Pathways (SSPs) Global Population and Human Capital Projections* (IIASA Working Paper WP-24-003). https://pure.iiasa.ac.at/19487. Available at: http://www.wittgensteincentre.org/dataexplorer.

Marois, G., & KC, S. (2021). *Microsimulation Population Projections with SAS. A Reference Guide*. Springer Nature.

Marois, G., Potančoková, M., & González-Leonardo, M. (2023). *QuantMig-Mic microsimulation population projection model.* [QuantMig Project Deliverable D8.2.]. International Institute for Applied Systems Analysis.

Marois, G., Sabourin, P., & Bélanger, A. (2019). Forecasting Human Capital of EU Member Countries Accounting for Sociocultural Determinants. *Journal of Demographic Economics*, *85*(3), 231–269. https://doi.org/10.1017/dem.2019.4

Potančoková, M., & Marois, G. (2020). Projecting the future births in the EU28 with fertility differentials reflecting women’s educational and migrant characteristics. *Vienna Yearbook of Population Research*.
